# Medical Treatment Following Violence Exposure in a National Sample of Children and Youth

**DOI:** 10.1001/jamanetworkopen.2021.9250

**Published:** 2021-05-12

**Authors:** David Finkelhor, Heather Turner, Deirdre LaSelva

**Affiliations:** 1 Crimes Against Children Research Center, University of New Hampshire, Durham, New Hampshire; 2 Department of Sociology, University of New Hampshire, Durham, New Hampshire

## Abstract

**Importance:**

Children and youth experience high rates of exposure to violence, which is associated with later poor physical and mental health outcomes. The immediate injuries and impacts from these exposures are often treated in emergency departments and medical offices.

**Objective:**

To characterize, using nationally representative data, the size and characteristics of the child and youth population being seen by medical authorities in the wake of violence exposure.

**Design, Setting, and Participants:**

The survey study used a representative sample of children and youth aged 2 to 17 years, from 2 waves (2011 and 2014) of the National Survey of Children Exposed to Violence, drawn from a mix of random digit dialing and address-based sampling. Interviews were conducted (1) over the phone with caregivers of young children or (2) directly with the youth aged 10 to 17 years. Data analysis was performed from September to December 2020.

**Main Outcomes and Measures:**

Violence exposures were assessed with the 53-item Juvenile Victimization Questionnaire, which had follow-up questions that asked about injury and going “to the hospital, a doctor’s office, or some kind of health clinic because of what happened.” Additional questions were asked about lifetime and past-year childhood adversities and current trauma symptoms using the Trauma Symptom Checklist and the Trauma Symptom Checklist for Young Children.

**Results:**

The combined 2-survey sample had 5187 children and youth who reported a lifetime violence exposure, of whom 45.6% (95% CI, 43.1%-48.2%) were aged 2 to 9 years, and 54.4% (95% CI, 51.8%-56.9%) were aged 10 to 17 years; 53.6% (95% CI, 51.0%-56.2%) were male. Based on the full sample of 8503 children and youth, 3.4% (95% CI, 2.6%-4.4%) had a violence-related medical visit at some time in their lives. The rate of past-year medical visits due to a violence exposure was 1.9% (95% CI, 1.2%-2.7%), equivalent to a point estimate of approximately 1.4 million children and youth. Of those with medical visits, 33.3% (95% CI, 23.1%-45.4%) were aged 2 to 9 years. Those with a past-year visit had higher levels of trauma symptoms (risk ratio, 1.71; 95% CI, 1.44-2.03) adverse childhood experiences (risk ratio, 2.55; 95% CI, 2.34-2.78) and multiple violence exposures (risk ratio, 3.91; 95% CI, 3.22-4.76) compared with the general sample of children and youth.

**Conclusions and Relevance:**

The estimated large number of violence-related visits with medical professionals offers an opportunity to address a source of frequent injury, and provide counseling and referral for a high-risk segment of the population to treat and prevent further physical and mental health and social consequences.

## Key Points

**Question:**

How many children and youth receive medical care in the US as a result of assault, abuse, or violence exposure?

**Findings:**

In this study of 8503 children and youth participants in the US National Survey of Children Exposed to Violence, 3.4% of the participants had a violence-related medical visit at some time in their lives and 1.9% in the past year; approximately one-third were under the age of 10 years. Those receiving treatment had more trauma symptoms, additional violence exposures, and more adverse childhood experiences than other children.

**Meaning:**

The estimated large number of violence-related medical visits offers an opportunity to provide referral for a high-risk segment of the population to prevent further physical and mental health and social consequences.

## Teaser

This survey study examines data from interviews with youth aged 10 to 17 years and caregivers of children aged 2 to 9 years to assess how many youth in these age groups have received medical care in the US as a result of violence.

## Introduction

Children and youth experience high rates of assault and violence, including family maltreatment, peer assault, sex crimes, and community violence.^1,2,3,4^ A portion of these exposures result in harms that prompt visits to health professionals, emergency departments (EDs), pediatricians, family physicians, and school health services.^5,6^

Much of the prior epidemiology of these exposures has been based on hospital ED data.^7^ For example, estimates from a systematic sample, the National Hospital Ambulatory Medical Care Survey, found that 340 000 children aged 0 to 17 years were treated in EDs in a typical year between 2000 and 2008 for violence-related injuries (a rate of 49 per 10 000), accounting for approximately 1% of all of pediatric ED visits for any reason.^8^

However, most of the ED-based epidemiological analyses were focused on older youth,^9,10^ or certain specific types of exposures such as sexual assault^11^ or bullying,^12^ and did not include health care settings beyond EDs.

Documenting medical visits from violence-exposed children to EDs and other services is important, not simply because it allows an estimate of the scope of the seriously affected. These visits also represent opportunities, if properly managed, to identify and intervene with children at potentially high risk for experiencing additional exposures. These children likely also belong to the ranks of those with mental health problems and high levels of other adversities.^13^ Health care professionals attuned to the needs of this violence-exposed population can take advantage of these visits with interventions that may reduce the physical and mental toll of the current as well as future exposures.^14^

## Methods

### Sample and Procedure

The analyses that follow use data from 2 of the National Surveys of Children’s Exposure to Violence, cross-sectional surveys that collected information about nationally representative samples of youth aged 1 month to 17 years in 2011 and 2014. The samples from each of the surveys were obtained from a mix of random digit dialing, address-based sampling, as well as targeted oversampling of households with children and youth, cell phone–only households, and underrepresented racial groups. Interviews began with an adult caregiver in each household to collect family demographic information. One child was randomly selected from all eligible children living in a household by sampling the child with the most recent birthday. Telephone interviews were conducted directly with youth aged 10 years and older about their experiences of violence, symptoms, and other topics. If the selected child was under age 10 years, proxy interviews were conducted with the caregiver “who was most familiar with the everyday experiences of the child.” Using the American Association for Public Opinion Research Response Rate #4 calculation, the survey response rates were 44.6% in 2011 and 24.1% in 2014.^15^

Sample weights adjusted for differential probability resulting from both this complex study design as well as variations within household eligibility and nonresponse by demographic characteristics. More information about the sample and weighting is available in prior publications.^1,16,17^ The analysis for the current study used pooled data from both the 2011 and 2014 surveys, focusing on children and youth aged 2 to 17 years, for a total sample size of 8503 (4232 aged 2 to 9 years; 4271 aged 10 to 17 years).

Interviews averaged approximately 50 minutes in length and were conducted in English or Spanish. Respondents who disclosed a situation of serious threat or ongoing abuse were recontacted by a clinical member of the research team, trained in telephone crisis counseling, whose responsibility was to provide them with contact information for support in their local community. All study materials were reviewed and approved by the University of New Hampshire institutional review board. Verbal consent was obtained from parents, and verbal assent was obtained from youth who were interviewed. All data used for analysis were deidentified. This study followed the American Association for Public Opinion Research (AAPOR) reporting guidelines.

### Measurement

#### Violence Exposure

Violence exposure was based on the Juvenile Victimization Questionnaire (JVQ), an inventory of childhood exposures^18,19,20^ that obtains reports on 53 forms of offenses against youth that covers 6 general areas: conventional crime, child maltreatment, peer and sibling abuse, sexual assault, witnessing and indirect violence exposure, and internet offense exposure. Follow-up questions for each screener item gathered additional information, including perpetrator characteristics, the use of a weapon, whether injury resulted, and to whom the episode was disclosed.

#### Injury and Medical Treatment

Follow-up questions about injury and medical treatment were asked in connection to 16 types of violence exposure that could potentially be associated with physical harm. The injury question read: “(Was your child/Were you) physically hurt when this happened? Hurt means you could still feel pain in your body the next day. You are also hurt when you have a bruise, a cut that bleeds, or a broken bone.” The medical treatment question was: “Did (your child/you) go to the hospital, a doctor’s office or some kind of health clinic because of what happened?”

#### Poly-victimization

The literature has identified a group of children who suffer from multiple kinds of violence exposure.^13,21^ These children appear to be in a sustained and multicontext condition of vulnerability and are substantially more likely than other children to experience physical harm and additional forms of ongoing violence exposure. Consistent with earlier research,^5,22^ the cutoff scores for poly-victimization were 5 or more exposures for children aged 2 to 5 years, 7 for children aged 6 to 9 years, 8 for those aged 10 to 13 years, and 9 for those aged 14 to 18 years.

#### Adverse Childhood Experiences

The study interviews included numerous questions and indexes from which to draw a large pool of adverse childhood experiences (ACEs) that fell within several content domains: abuse, family instability, interpersonal loss, parent psychological disorder, environmental threat, and economic stressors. In previous research,^23^ we distilled 40 adversities into a set of 15-item inventories that best predicted adverse outcomes for each age group.^23^ Based on those analyses a high-ACEs group was demarcated by the presence of 5 or more adversities among children aged 2 to 9 years or 7 or more adversities among the those aged 10 to 17 years.

#### Trauma Symptoms

Trauma symptoms were assessed using 24 items from the Trauma Symptom Checklist (TSC)^24^ designed for youth aged 10 years and older and 26 items from Trauma Symptom Checklist for Young Children (TSCY), completed by caregivers of children aged 2 to 9 years. Both scales assess children’s responses to unspecified traumatic events in different symptom domains, including depression, anxiety, anger, posttraumatic stress, and dissociation. Respondents were asked to indicate how often they (or their child) had experienced each symptom within the last month. The TSC and TSCY have demonstrated good test-retest and internal consistency reliability and good concurrent validity in clinical and population-based samples.^24,25^ We retested our selected items for this study, chosen to represent the domains and maximize internal consistency, and the alpha coefficients remained high at 0.93 for the TSCC and 0.86 for the TSCYC. A summary measure of all items was constructed for each age group: 2 to 9 years and 10 to 17 years. We constructed a high-trauma symptom indicator in order to identify cases of potential clinical significance. High-trauma symptoms were defined as the top decile of the summary measure for each age group.^23^

### Statistical Analysis

The logistic regression analysis of demographic and episode characteristics was conducted using the sample of all 5187 youth with violent episodes reported by respondents. The outcome variable compared violence-exposed youth who had a medical visit with violence-exposed youth who did not. The outcome of having a medical visit was regressed on several variables including child's race, sex, age, parents' educational attainment, family structure, past-year violence exposure categories, whether there was reported injury, whether the police knew about the offense, whether the child's teacher knew about the offense, and whether the perpetrator was an adult. Survey weights were applied. For the bivariate risk ratio analysis of current symptoms, lifetime adversities, and current poly-victimization status, the analysis used only past-year medical visits to make sure that the health care visits were contemporaneous with or subsequent to these vulnerability measures. The comparison group for the vulnerability measures was all other children and youth without a past-year visit, whether violence-exposed or not.

The significance threshold was set at *P* < .05 using 2-sided Pearson χ^2^ test. Data analyses were conducted using Stata/SE version 16.0 (StataCorp) from September to December 2020.

## Results

The combined 2-survey sample consisted of 8503 children aged 2 to 17 years, 54.0% (95% CI, 51.9%-56.0%) aged 2 to 9 years, and 46.0% (95% CI, 44.0%-48.1%) aged 10 to 17 years; 51.2% (95% CI, 49.1%-53.2%) were male. The analysis of variables associated with a medical visit was conducted on the sample of 5187 children who reported a lifetime violence exposure, of whom 45.6% (95% CI, 43.1%-48.2%) were aged 2 to 9 years, and 54.4% (95% CI, 51.8% to 56.9%) were aged 10 to 17 years; 53.6% (95% CI, 51.0%-56.2%) were male.

Out of the whole sample, 3.4% (95% CI, 2.6%-4.4%) of the children and youth had a violence-related medical visit at some time in their lives. The rate for such a medical visit for the past year was 1.9% (95% CI, 1.2%-2.7%). Based on a US child and youth population of 73.75 million, the mean for the period 2011 to 2014, this would suggest a point estimate of approximately 1.4 million youth with violence-related medical visits for a 12-month period in that interval. Of those with medical visits, 33.3% (95% CI, 23.1%-45.4%) were aged 2 to 9 years.

The majority of the medical visits (71%) were for peer violence, and 23% were for sexual assaults. Parental child maltreatment composed 1% of the total. Sexual assaults by adults were the kind of exposure most often to result in a medical visit (20%) (Table 1). For all violence exposures, medical visits were less frequent in comparison with disclosures to police and teachers.

**Table 1.** Percentage of Violence-Exposed Children and Youth Who Reported Injury, Police Contact, Their Teacher Knowing, or Medical Visit^a^

Violence Type	Youth, No. (unweighted)	Weighted %			
		Injury	Police contact	Teacher knows	Medical visit
Sexual assault by adult	90	20.8	44.0	39.6	19.6
Gang assault	226	38.8	20.2	54.5	9.0
Custody interference	269	6.4	39.3	25.6	8.7
Kidnap	108	1.1	45.2	25.7	8.5
Assault with weapon	697	43.2	13.2	32.0	6.0
Sexual assault by peer	150	11.7	10.9	24.0	6.0
Bias attack	192	33.3	15.8	59.2	5.6
Assault without weapon	2029	35.6	10.1	41.4	4.4
Rape	189	12.0	14.3	20.4	4.2
Physical abuse	653	34.4	17.3	20.1	4.1
Genital assault	826	30.2	4.9	24.7	3.9
Robbery	1220	8.1	4.5	33.7	2.0
Date violence	107	36.7	7.4	9.3	1.1
Assault by peer/sibling	3996	16.1	3.0	18.5	1.0
Bullying	2182	6.6	2.6	25.4	1.0
Any violence	5187	27.4	10.2	38.2	4.5

^a^ Table data presented in descending order by percentage with medical visit.

Although most of those with medical visits had injuries, 14% (95% CI, 8.1%-22.9%) of the visitors reported no injury. This group was too small for separate analysis, and no details were gathered about what prompted the visits among the noninjured. They could have involved matters like forensic documentation, assessment for possible injuries that were not confirmed, and seeking medical consultation about mental health and psychosocial issues.^26^

Logistic regression with demographic and episode characteristics showed that, aside from the obvious factor of sustaining an injury, the factor with the strongest association with a medical visit was that the police knew about the episode (Table 2). The association could be bidirectional. Reports to police may prompt medical visits due to police recommendation or for purposes evidence gathering. It could also be that medical visits prompt reports to police because of practitioner concern or legal obligation under mandatory reporting laws. Teacher awareness of the offense was also associated with increased medical attention. Age, sex, and family education level were not associated with medical visits once injury and report to authority were controlled. There was, however, a significant association for a child living in a nonparental household (log odds ratio for other adult category, 3.3 [95% CI, 1.6-7.1]; *P* = .002) (Table 2).

**Table 2.** Logistic Regression of Violence-Exposed Children and Youth Receiving Any Medical Treatment Following Exposure vs No Treatment on Demographic and Exposure Characteristic Variables

Received medical treatment	Log odds ratios (95% CI)
Race	
White	1 [Reference]
Black	0.6 (0.3-1.3)
Hispanic	0.7 (0.4-1.3)
Other^a^	0.7 (0.2-2.6)
Sex	
Female	1 [Reference]
Male	0.8 (0.5-1.2)
Age group, y	
2-9	1 [Reference]
10-17	0.9 (0.5-1.5)
Location	
Urban	1 [Reference]
Rural	0.9 (0.5-1.4)
Parent education	
Less than high school	1 [Reference]
High school	1.9 (0.7-4.8)
College	1.0 (0.4-2.6)
Grad school	1.4 (0.5-3.8)
Family structure	
Both parents	1 [Reference]
Single parent	1.2 (0.7-2.0)
Parent/step-parent	0.8 (0.4-1.8)
Other adult	3.3 (1.6-7.1)^b^
Violence type	
Conventional crime^c^	1.5 (0.8-3.0)
Parental maltreatment	1.0 (0.5-1.9)
Peer abuse	1.8 (0.8-3.7)
Sexual assault	1.7 (0.9-3.3)
Any injury	10.7 (5.0-23.0)^d^
Adult perpetrator	1.2 (0.6-2.5)
Police know about exposure	7.1 (4.3-11.8)^d^
Teacher knows about exposure	2.9 (1.6-5.3)^d^

^a^ Other racial category includes Alaskan Native, American Indian, Asian, or mixed race.

^b^ *P* = .002.

^c^ Conventional crime includes robbery, assault with weapon, assault without weapon, kidnapping, or bias attack.

^d^ *P* < .001.

Children and youth with medical visits in the past year had higher than normal levels of general vulnerability, as indicated by adversity and symptom profiles. In the medical visit group, 28% had high levels of trauma symptoms, 87% had high adverse childhood experience scores, and 41% had unusually high levels of different kinds of violence exposures (poly-victimization).^27^ The risk ratio for these vulnerabilities in the medical visit group (in comparison with the other children in the sample) was 1.71 (95% CI, 1.44-2.03) for trauma symptoms, 2.55 (95% CI, 2.34-2.78) for high ACES, and 3.91 (95% CI, 3.22-4.76) for poly-victimization.

## Discussion

An estimated 1.4 million children and youth visited medical practitioners in the course of a year for help related to a violence exposure, according to this national survey. This is larger than estimates based on hospital ER visits, and likely reflects in part the reality that non-ER health settings receive considerable usage from violence-exposed children and youth. These non-ER settings may include urgent care facilities, private practices, and the offices of school health personnel. Whereas hospital ER studies generally focus on adolescents exposed to violence, perhaps assuming them to be the high-risk group, the present study revealed considerable use by children aged 2 to 9 years. It is important that studies of medical response to crime, violence, and abuse recognize the full developmental spectrum of exposures.

It should be kept in mind that the medical care visits represent a small portion of violence-exposed children and youth and also only a minority of those with injuries. But they include many of the most serious exposures and most vulnerable children—sexual assaults, kidnappings, gang assaults, and aggravated assaults. They also disproportionately include children with high levels of previous violence exposure, children with many childhood adversities, and children manifesting mental health symptoms.

The medical visits likely fulfill several functions: treating injuries as well as providing counseling about the underlying safety issues, medical documentation to support criminal investigations or parental complaints, and referrals to behavioral health and family services. The forensic documentation role is supported by the finding that police contact is associated with medical care, even controlling for injury and type of violence. It is likely that some of the association with police contact may also be health personnel making referrals to the police based on their examination and abiding by mandatory reporting statutes.^11^

The children and youth who make medical visits for violence-related causes are a high-risk group. Almost half have had multiple different kinds of other violence exposures. More than half had an extreme history of ACEs more generally. Almost a third had clinical levels of trauma symptoms. This means they are a population in need of more than simple treatment or documentation of injury.

### Limitations

This study and its design have limitations that need to be kept in mind. Information about visits to medical care professionals were gleaned from 2 different groups, caregivers in the case of children aged 2 to 9 years, and youth themselves in the case of children aged 10 to 17 years. Caregivers in particular may not be aware of all episodes and may underreport those inflicted by themselves or other family members. The omission of children under age 2 years is a significant limitation, because very young children have higher rates in clinical samples of medically identified injuries due to parental child maltreatment. Moreover, because many of such abuse-related injuries to younger children have their cause concealed when presented for treatment, it may be that caregivers also concealed information about them in the survey, despite its confidential design. Also, some of the episodes occurred years in the past and may be subject to memory distortion. The youth respondents in particular are likely to underreport episodes occurring early in life, before the horizon of their own personal memories.

In addition, the study has limited information about the details of the medical visit, such as the profession or institutional location of the clinician, and whether it was for diagnosis, treatment, or forensic purposes. Similarly, no information is available about the nature or seriousness of the injuries, beyond that they involved a bruise, a cut that bleeds, broken bone, or pain that lasts into the next day.

## Conclusions

This survey study found that a considerable proportion of children and youth exposed to violence receive medical care. Several approaches are warranted with children and youth presenting with possible violence exposure. A first obvious need is to try to ascertain that the injuries are inflicted, since this may well be masked. Probes need to be asked about safety and current risk on topics such as gang involvement, family maltreatment, chronic bullying, weapon ownership and access, and patterns of alcohol and substance usage. In addition, it is important to assess for other adversities, prior violence exposures, and behavioral symptoms.^14^ There are some evaluated models for intervention, especially in EDs and hospital-based settings. They typically involve motivational interviewing to help victims consider the possible need for intervention and then referrals to resources for help.^28,29,30,31,32^

Our finding about considerable violence-related medical visits from preteenage youth raises the potential for early intervention. These require treatment models that work with parents. Several avenues for evaluation and treatment ought to be part of the typical assessment. These include signs of emotion dysregulation and symptoms that may be putting the youth at risk, problems in the parent-child relationship that are preventing adequate supervision, or ineffective or abusive behavioral management. Also to be considered are dangerous school and neighborhood environments. Practitioners should be prepared to provide information about access to prevention education skills training. There is increasing availability of safety skill programs that use digital technology to provide education for those at risk for violence exposure^33,34^ that may prove invaluable in these contexts. But facilitating such evaluation and referral requires adequately trained behavioral health and social work professionals working in the practices and emergency departments where such children are being seen.
